# Injectable Hydrogels for Chronic Skin Wound Management: A Concise Review

**DOI:** 10.3390/biomedicines9050527

**Published:** 2021-05-10

**Authors:** Mazlan Zawani, Mh Busra Fauzi

**Affiliations:** Centre for Tissue Engineering and Regenerative Medicine, Faculty of Medicine, The National University of Malaysia, Kuala Lumpur 56000, Malaysia; p109645@siswa.ukm.edu.my

**Keywords:** injectable hydrogels, skin wound, diabetic foot ulcer, advanced dressings

## Abstract

Diabetic foot ulcers (DFU) are a predominant impediment among diabetic patients, increasing morbidity and wound care costs. There are various strategies including using biomaterials have been explored for the management of DFU. This paper will review the injectable hydrogel application as the most studied polymer-based hydrogel based on published journals and articles. The main key factors that will be discussed in chronic wounds focusing on diabetic ulcers include the socioeconomic burden of chronic wounds, biomaterials implicated by the government for DFU management, commercial hydrogel product, mechanism of injectable hydrogel, the current study of novel injectable hydrogel and the future perspectives of injectable hydrogel for the management of DFU.

## 1. Introduction

The skin is the body’s first defence mechanism as it acts as a shield from external pathogens and it initiates Vitamin D synthesis, thermal regulation and hydration. As the largest organ in the body, the skin plays a vital role in maintaining the physiological haemostasis of the body hence, chronic skin damage may be lethal if left untreated. Wound healing can be delayed by chronic conditions such as diabetes mellitus or peripheral vascular disease [1].

Wound, defined as an injury usually occurs within the skin, connective tissues or the mucus membrane may result in the structural and/or functional defects of the organs [2]. A wound can be categorised into two main types namely acute and chronic wounds. Traumatic or even surgical wound is classified under acute wounds as it normally surpasses the wound healing phase and repair arrangement within the expected period [3]. The regulation of protease in this category is maintained to assist proliferation, hence regulating extracellular regeneration. In contrast with chronic wounds, this type of wound involves a much more distorted healing process that can be classified as ulcers including all the others as pressure, venous, arterial, vascular and DFU. These chronic wounds demonstrate stalled inflammatory phase resulting in the development of biofilm, bacterial clusters, and elevation of protease at the site of the wound. This could be due to the extended period of pro-inflammatory cytokines (interleukin-1; IL-1) and tumour necrosis factor (TNF) accumulation [1,4]. The domination of protease above the inhibitors leads to extracellular matrix destruction, thus accelerating the proliferation and inflammation phase. This process then triggers reactive oxygen species to rise hence resulting in premature cells and defective extracellular matrix proteins [5].

Skin ulcers are often being related to diabetes, which is defined as the loss of the epithelium lining continuity, a dry type of stratified squamous epithelium (which causes the underlying tissue surface to be bare open). These ulcers breach the skin protective barrier that is keratin and its keratinocytes known as the anchor of the skin membrane [2]. In response to the injury, the body will endeavour to repair the damage locally and systemically according to the depth of the injury inflicted on the tissues. The healing process manifests an astonishing cellular mechanism distinctive in nature as every unique cell found in the tissue is capable of holding different aptitudes to replicate, divide and differentiate [2,6]. The interaction of cells, growth factors, and cytokines are involved in wound repair, which is a crucial part of the healing process.

## 2. Wound Healing Phase

**Figure 1:**
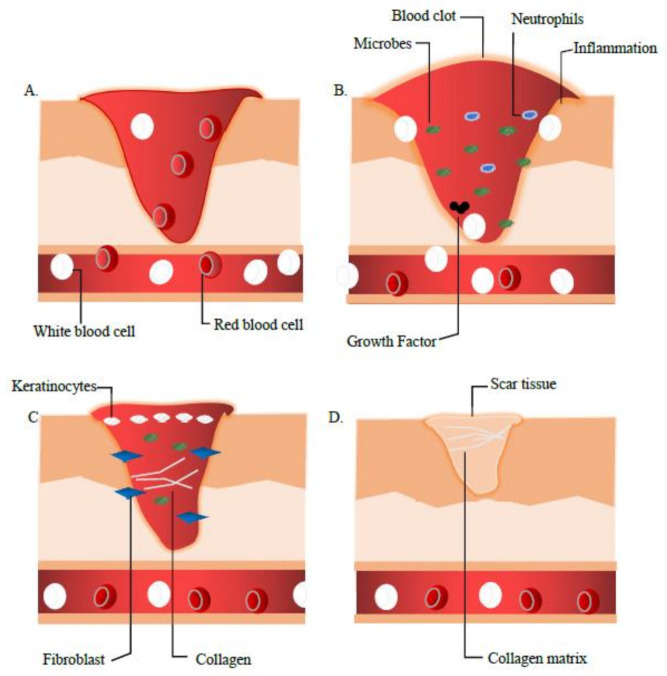
The wound healing process myriad which concludes the 4 continuous phases (**A**) *Homeostasis,* (**B**) *Inflammation,* (**C**) *Proliferation, and* (**D**) *Remodelling.* During these 4 stages, prior to the injury, the blood platelets are activated to form a blood clot, which also plays a role in leukocyte recruitment. Then as shown in (**B**). Neutrophils and macrophages, removes debris and fight infection (bacteria, dead cells, pathogens). (**C**). Here, the angiogenesis process begins where fibroblast migrate and proliferate. In the remodelling phase, the collagen matrix is formed to restore extracellular matrix, which turns into mature scar tissue (granulation tissue).

The wound healing process involves 4 major phases that are (a) Haemostasis, (b) Inflammation, (c) Proliferation and Migration and (d) Remodelling as shown in Figure 1.

### 2.1. Phase 1: Haemostasis

As the first combat of cell repair, during this phase, the platelets are activated, aggregate and adhere to the damage and defect squamous endothelial to conserve the haemostasis via coagulation. As the phenomena inaugurate, fibrin from the fibrinogen forms an embolus that acts as an impermanent extracellular matrix (ECM). The activated cells (platelets, neutrophils, monocytes) emancipate proteins and growth factors such as platelet-derived growth factor (PDGF) and transforming growth factor β (TGF-β). Alteration of the haemostasis phase was observed in diabetes mellitus (DM) patient by the reduction of hyper-coagulation and fibrinolysis [7].

### 2.2. Phase 2: Inflammation

This phase is inaugurated by neutrophils, mast cells and macrophages causing the production of inflammatory cytokines (interleukin 1 (IL-1), tumour necrosis factor-alpha (TNF-α), interleukin 6 (IL-6), and interferon-gamma (IFN-γ) besides the growth factors of platelet-derived growth factor (PDGF), transforming growth factor β (TGF-β), insulin-like growth factor 1 (IGF-1), and epidermal growth factor (EGF) that are the main essentials in the wound healing process [8]. Xiao et al. reported the presence of cytokines imbalance in diabetics patients that could lead to the alteration in the wound healing process. The modified cytokine distribution pattern has led to the diminution of their function hence causing the wound to be prone to infection [7].

### 2.3. Phase 3: Proliferation

The migration and proliferation processes begin in this phase including wound contraction to angiogenesis action. These actions include the restoration of oxygen supply, the genesis of extracellular matrix (ECM) protein, vitronectin and collagen, proliferation and migration of fibroblast and keratinocytes that are the key essentials for the recovery of the integrity and functionality of the tissues [8]. Hyperglycaemic condition in DM patients altered the functionality of fibroblast and keratinocytes to migrate and proliferate. Therefore, the abnormal cells cause the stagnation of angiogenesis, which eventually affects the healing process [9].

### 2.4. Phase 4: Remodelling

The modelling phase is in action 7 days after the injury and can last up to 6 months. The collagen III is synthesised and was replaced with collagen I to restore the ECM. The wound becomes resistant, and the mature scar tissue is formed (granulation tissue) leading to scar tissue [7,10]. Alteration of the fibroblast functionality in diabetics patients causes the deformation of the wound closure. Maione et al. [11] in a study stated that this might be caused by the unresponsive action to the transforming growth factor β and the abnormal ECM production.

## 3. Chronic Wound Primary Contributor

There are a number of chronic wound aetiologies burdening the healthcare institution [12]. Patients with chronic diseases such as diabetes and obesity are the two major contributors to the increase in the number of chronic wounds worldwide. This type of wound is commonly associated with comorbidities making it strenuous to trace it as a disease [12].

Diabetes mellitus (DM) is a chronic disease that occurs as the result of the reduction of insulin hormone in the body or when the body itself is not able to utilise the insulin effectively. Insulin, a hormone secreted from the pancreas assists the regulation of blood glucose in the body. DM is also classified as a metabolic disease and is 1 of 4 priorities for non-communicable disease, which have the highest impact on the health and socioeconomic burden worldwide [13]. The high prevalence of diabetes in adults increases the risk of foot problems mainly due to neuropathy and peripheral arterial disease [14]. DFU is commonly present from the distal level to the ankle among diabetic patients [15] in which diabetic peripheral neuropathy affects up to 50% of diabetic patients without exhibiting any symptoms [7]. Each year, approximately one million amputations are performed on diabetic patients around the world [16,17]. DFU requires special care and coordination ideally from a multidisciplinary foot care team.

Diabetic foot diseases (DFD) are the result of uncontrolled diabetes and are the main complications that occur in DM patients contributing to neuropathy, peripheral vascular disease, high foot plantar pressure, foot trauma and atherosclerosis, hence, increase in morbidity [17,18,19]. The combination of neuropathy or peripheral vascular disease will eventually lead to the development of diabetic ulcer. DFU frequently involves full-thickness skin loss, which then progresses to the deterioration of the bone, joint, and soft tissues [18]. These will inflict complications to the diabetic patients hence amputations are performed in >50% of the patients. The wound healing phase is mainly compromised during this complication that could expose the ulcers to infections, stagnant proliferations, stall inflammatory phase, prolonged angiogenesis and impaired foot deformity. These events will eventually lead to surgical debridement and amputation of the defect area to obviate further systemic sepsis [20,21].

### 3.1. Socioeconomic Burden of Chronic Wound

The annual chronic wounds have become more common over the last decade owing primarily to the growing global geriatric population and the rising prevalence of obesity and diabetes (4–10%) [15,22,23,24]. Diabetes and obesity can increase the overall incidence and complexity of wounds such as infections, ulcers and surgical wounds, necessitating treatment and resulting in exorbitant medical expense with an estimated prevalence of 15% to 25% [15]. With an estimated treatment cost of $28.1 billion to $96.8 billion for approximately 8.2 million Medicare beneficiaries, the second highest expenses are for DFU wound care [12]. An average of 252 million pounds is allocated yearly for the treatment of DFU worldwide in which economy statistic suggested that annual market of wound care products is expected to outstretch to a total of $15–22 billion by 2024 [12].

Thorough classification of DFU severity and stage is vital for the management of diabetic foot disease as meticulous management is required as well as on DM disease [7]. An adequate wound care management is much needed for this type of ulcers hence, the regiment mainly focused on sepsis control, alleviation of pressure, optimal perfusion and adequate wound debridement [7]

### 3.2. Current Treatment of Diabetic Foot Ulcer

The Malaysian government primarily through Ministry of Health implicated various biomaterials for the management of DFU including advanced dressings such as hyaluronic acid gel, hydrocolloid dressing, alginate dressing, hydrofiber dressing, iodine-impregnated dressing, foam adhesive dressings and protease-modulating matrix dressing. Silver-impregnated dressings are only used in wounds with or at risk of high bioburden or local infection [25]. The mechanism and indications of these advanced dressings are similar to hydrogels as the purpose was to absorb wound exudates to maintain a wound haemostasis environment in promoting wound healing and skin regeneration [26].

### 3.3. Contraindications and Complications of Current Treatment

On the other hand, the hypoxic atmosphere of these dressings can cause wound healing to be delayed or hampered, hence increasing the risk of microbial infection. Alayi et al., stated that the acrylics dressings could be difficult to remove as it possesses a low capacity to absorb elucidates [27]. In addition, an allergic reaction may develop in the hydrocolloid dressings aid in the autolytic debridement. This can cause microbial infection to the wound, which can raise difficulty during the removal process [25,26,27]. Furthermore, frequent dressings replacement is required in foam adhesive dressing that is known to induce peri-wound maceration and causes discomfort towards the patients [20]. As for hydrofiber and alginate dressings, both need a support of a secondary dressing. Alginate is frequently confused with pus or slough on the wound and may induce allergic reaction [25,26,27]. The benefits and complications of the current dressing treatment are summarized in Table 1.

## 4. Tissue Engineering Advancement

With technological advancement, other series of therapies for DFU have been implemented such as the development of skin substitutes, negative pressure wound therapy, hyperbaric oxygen, fabrication of novel wound dressings that implicate growth factors, and the use of tissue-derived biomaterials. In tissue engineering, biomaterials play a vital role as a provisional bioscaffold for tissue repair and regeneration [28,29,30,31].

### 4.1. Tissue-Derived Biomaterials

Tissue-derived biomaterials utilise both tissue engineering and regenerative medicine to accelerate and assist diabetic wound healing. As it is derived from tissue, these biomaterials mimic the native of our soft tissues hence, nurturing a biocompatible microenvironment [32]. These biomaterials are harvested from cadaveric tissue allografts, placenta, submucosa of the small intestines, and algae [32,33]. The epidermis of the cadaveric human skin was removed to fabricate or construct the cadaveric allograft hence, abstaining from biological rejection [33]. Another source of these materials is human amnion and chorion membrane (dehydrated), the layer of epithelial cells, basement membrane and avascular connective tissue matrix on which was scientifically proven to accelerate wound healing and showed elevated wound closure compared to standard wound management. A study performed by Zheng et al. by utilising cryopreserved microamnion on diabetic-induced mice showed that the biomaterials accelerated wound healing and increasing growth factors regulation and neovascularisation [34]. These biomaterials are excellent in mimicking the ECM scaffolds promoting high biocompatibility for wound healing purposes.

### 4.2. Hydrogel-Based Biomaterials

Engineered hydrogels have the advantage of being able to alter their properties and have more defined ingredients than tissue-derived matrices. They are usually designed and engineered to mimic the ECM found in natural soft tissues [35]. These biomaterials can be made as high-water-content hydrogels, sponge and patch structures or other architectures. They can also be crosslinked, dehydrated, freeze-dried or electrospun [36,37,38]. Poly-N-acetyl Glucosamine (pGlcNAc) is a microalgae-derived matrix that has been approved by the Food and Drug Administration (FDA) for the treatment of DFU [39]. However, the most favoured material in the development and fabrication of hydrogel for DFU management is fibrin. Fibrin has been proven to accelerate angiogenesis and regulate the wound inflammation process hence facilitating the wound healing process. [40].

## 5. Commercialised Hydrogels for Diabetic Foot Management

DFU is the primary cause of DM complication that often causes a number of medical complications. Therefore, advancement in hydrogel-based biomaterial has shed some light to provide a better quality of life for the DFU patients. One of the advancements is the newly commercialised Apligraf^®^ and Leucopatch^®^ cellular hydrogel-based biomaterials that have been approved by the FDA for the management of DFU [41,42]. Apligraf^®^ is a bilayer skin substitute bioengineered with living cells in helping to accelerate DFU wound healing. Apligraf^®^ is composed of the dermal and epidermal layer that is made of bovine collagen type 1 incorporated with human dermal fibroblast. These composites promote suitable hydration and protein to accelerate wound healing as well as to support cell attachment [42]. Meanwhile, Integra is a collagen-glycosaminoglycan bioscaffold hydrogel that has been approved in the market and has been applied in the clinic for fibroblast and endothelial invasion and capillary growth. Integra can support epithelialisation in the absence of vascularisation [43].

As a consequence of the accessibility of skin tissue and its inert capacity to regenerate, skin wound healing has long been a subject of regenerative medicine [44]. Immediate full-thickness skin wound management is a realistic approach to improving the healing rate and minimizing the risk of complications. Hence the tissue engineering method was applied to combat these difficulties by shedding light on a novel approach of developing bioinert injectable hydrogels. The unique characteristics of the hydrogel-based skin substitutes have attracted immense attention as they are able to mimic the skin extracellular matrix and its microenvironment [45].

## 6. Development of Injectable Hydrogel for Diabetic Foot Management

As a consequence of the accessibility of the skin tissue and its inert capacity to regenerate, skin wound healing has long been a subject of regenerative medicine [44]. Immediate full-thickness skin wound management is a realistic approach to improve the healing rate and minimising the risk of complications. Hence, the tissue engineering method was applied to combat these difficulties by a novel approach to developing bioinert injectable hydrogels. The unique characteristics of the hydrogel-based skin substitutes have attracted immense attention as they are able to mimic the skin ECM and its microenvironment [45].

## 7. Development of Injectable Hydrogel for Diabetic Foot Management

Hydrogels are a three-dimensional (3D) network of crosslinked polymers, predominantly water based. The hydrophilicity of these hydrogels makes them ideal to mimic the ECM as it is considered non-toxic, biocompatible, and self-degradable [46,47]. Several different types of hydrogels are categorised based on the materials used such as natural or synthetic hydrogels or based on the synthesis methods namely physical or chemical crosslinking. Hydrogels hold unique characteristics for study including a high degree of flexibility due to the versatile chemical and physical properties to develop the best hydrogel for a given application. The ability of the hydrogel to shape swollen 3D networks allows it to diffuse molecules and cells, but the resemblance to ECM makes them appealing for biomedical and tissue engineering applications [48].

There are a variety of biomaterials that can be used as matrices for wound healing purposes and can be categorised as either natural or synthetic. The most utilised natural biomaterials for wound management are collagen, gelatine, hyaluronic acid, alginate, chitosan, fibrin, cellulose and silk fibroin [49,50,51,52,53,54,55,56]. They are substantially discovered in the ECM arrangements with properties of bioinert, biodegradable, and possess strength and elasticity that are applicable for the use of tissue engineering and cell culture [45,46,47,48].

### 7.1. Mechanism of Injectable Hydrogel

A number of mechanisms have been utilised in the process of developing injectable hydrogels. However, the crosslinking methods are not only significant on the polymerisation of this injectable biomatrix, but also play an important role in synthesising hydrogels. Although the crosslinking process is the same, the main difference in the preparation of injectable gels and in situ forming gels is the flexibility in the regulation of the gelling kinetics. The sol-gel transformation of an injectable hydrogel should be managed to happen within a given time period. Injectability or mass transfer and bulk gel moulding can be affected by faster or slower gelation kinetics. Table 2 summarized the crosslinking methods utilize in the fabrication of injectable hydrogel.

**Figure 2:**
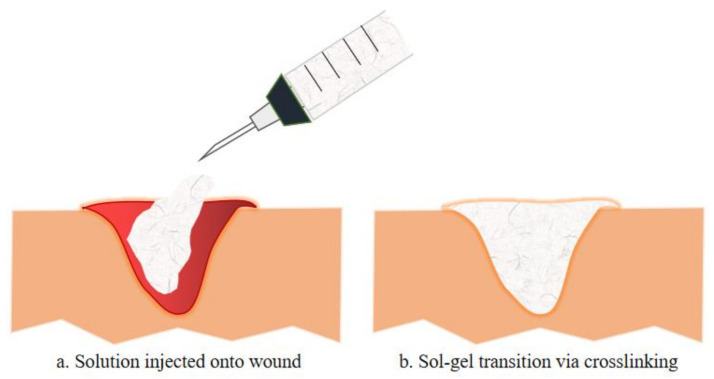
The exemplify of injectable hydrogel injected onto a wound is illustrated in a. whereas b. illustrates the sol-gel transition of the injectable hydrogel with the incorporation of therapeutic compositions, either by chemical or physical crosslinking. Such injectable hydrogels formed in situ have been used to deliver various therapeutic cells or biologics (e.g., growth factors, chemokines for modulating the function of endogenous cells) to promote tissue regeneration.

Injectable hydrogels for tissue engineering applications utilise several natural polymers, synthetic polymers, peptides, and proteins. These building blocks can be crosslinked physically or chemically while being incorporated with cells, tissues or biological materials [57]. Figure 2 illustrates the schematic diagram of sol-gel transition, a mechanism of injectable hydrogel that occurs by the interaction of the basic building block with the precursor (crosslinking agents). As shown, the mixture of the polymer and the based or therapeutic agents is injected onto the wound in a solution manner and polymerise once administered via crosslinking reaction [57]. This transition in viscosity of the solution as it turns into a gel form can be calculated by rheology test. Furthermore, this polymerisation method often utilised various external stimuli such as changes in temperature, pH, electromagnetic field or even enzyme and lights conditions. Chemical crosslinking can be divided into multiple reactions such as Schiff base reactions and Diels-Alder reactions, while physical crosslinking involves hydrogen bonding, van der Waals forces, π-interactions and hydrophobic interactions [58].

External factors such as temperature, pH, electric/magnetic fields, light, biomolecular species (such as enzymes) and others can affect and control the physical crosslinking, morphology, and properties of the injectable hydrogels. These stimuli-responsive hydrogels can be promising “smart” biomaterials for tissue engineering and disease therapy. The most widely published studies are on temperature-responsive hydrogels. A sol-gel transformation at a lower critical solution temperature is usually used to shape physical crosslinking [57].

#### 7.1.1. Physical Crosslinking

Physical crosslinking, one of the crosslinking reaction mechanisms used to make hydrogels, is caused by non-covalent interactions such as electrostatic interactions between two oppositely charged ions (such as ionic bonds, hydrogen bonds, and others), hydrophobic interactions, -interactions, and van der Waals forces such as dipole-dipole interactions and London dispersion forces. Furthermore, without the use of a crosslinker or catalyst, some injectable hydrogels can be made by physically self-crosslinking through interactions such as host-guest interactions [57,58,59]. Moreover, various external stimuli often influence crosslinking-induced gelation. The following is a study of the injectable hydrogels that have been recently produced using various physical crosslinking techniques.

#### 7.1.2. Chemical Crosslinking

Click chemistry or chemical crosslinking is highly efficient and possesses rapid crosslinking ability. The most commonly utilised chemical is Azide-alkyne catalysed from copper, but despite its outstanding chemistry in gel formation, it may be toxic to cell hence limiting its application for cell delivery and encapsulation. There are also many other chemicals or click reactions that are being utilised for crosslinking purpose including thiol-ene, diels-alder and oxime reactions [57,58,59].

### 7.2. Development of Injectable Hydrogel for Diabetic Ulcer Management

Several mechanisms need to be taken into consideration during the development of these injectable hydrogels in order to combat the complication of DFU. Several in vivo studies stated complex molecular mechanism that could hinder wound healing in diabetic-induce animals including sustained pro-inflammatory cytokines, impede angiogenesis reaction, vascular drawback, impaired dermal skin cells migration and proliferation [60].

Chronic wounds are often stalled in the inflammatory phase of wound healing causing severe persistent complications that could lead to amputation. In a study, Lee et al. used an electrospinning technique to create a nanofibrous collagen/poly-D-L-lactide–glycolide (PLGA) scaffold membrane. This scaffold could be applied to diabetic wounds and filled with medications to allow for long-term Glucophage release and wound healing [61]. In another study, an in vivo study in female Wistar rats demonstrated the ability of AgNP-loaded hydrogels to minimise wound size compared to injuries, encouraging histological changes in the healing tissue over the course of wound healing, as in earlier production and maturation of granulation tissue [62].

**Figure 3:**
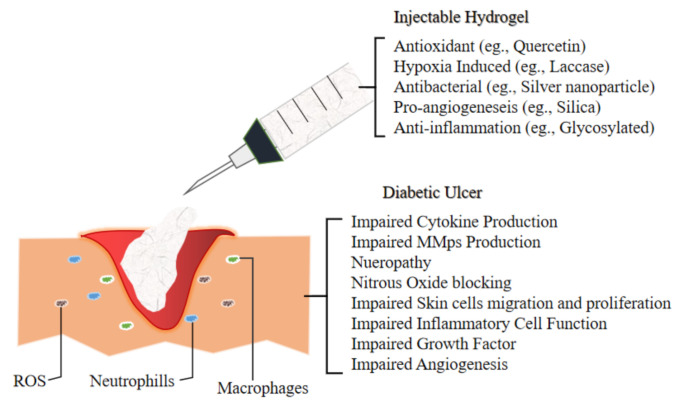
Illustrate the environment of Diabetic Ulcer and the impairment which lead to its slow recovery. Hence the development of novel injectable hydrogel for the management of Diabetic Foot Ulcer are incorporating various materials which exert antioxidant (to combat ROS), hypoxia induced, antibacterial (to overcome bacterial infection), pro angiogenesis (to accelerate wound healing by promoting new vessels for better blood flow) and anti-inflammation mechanism as inflammatory cells in the ulcer elevate to reactive oxygen species (ROS) level, hence ensued in extracellular matrix injuries as well as premature decrepitude of dermal cells.

The next section will discuss the future application of novel injectable hydrogel for diabetic ulcer including the development of thermosensitive injectable hydrogel, sustained-release or tissue-specific hydrogel. In another study, a team of researcher incorporated multi-active ingredients which are stated in Figure 3 that demonstrate antibacterial and anti-inflammation properties to diminish bacterial-induced inflammation and support as well as accelerating the wound healing process of chronic diabetic ulcer.

#### 7.2.1. Development of pH-Sensitive Injectable Hydrogel

The development of pH-sensitive injectable hydrogels has also been extensively studied. In a study by Qu et al., pH-sensitive and electric field responsive hydrogels were prepared using antibacterial and conductive chitosan-graft-polyaniline (CP) copolymers and oxidised dextrans (OD, crosslinker) via Schiff base reaction for the use as smart drug delivery systems. Amoxicillin and ibuprofen (model drugs) were loaded in the CP/OD hydrogels and the release rate of the drugs was increased by increasing the electric field voltage. The hydrogels showed pH-dependent degradation and morphology, good cytocompatibility, and the release regulated by pHs/electric fields were suggested as ideal biomaterials for smart drug delivery [63].

#### 7.2.2. Development of Thermosensitive Injectable Hydrogel

A group of researchers incorporated a combination of chitosan, collagen and β-glycerophosphate into their hydrogels. 3D mesenchymal stem cell sphere was then added to enhance wound healing activity by exerting the paracrine and neovascularise effects. These hydrogel mixtures are thermosensitive and polymerise when in contact with body temperature via physical crosslinking by filling the defect area despite the shape and depth. This biomatrix showed to exert an excellent therapeutic effect compared to other control groups. This technique was then examined by the elevation of cells attachment and proliferation for further investigation for the future use of chronic and venous wound management [64].

#### 7.2.3. Development of Sustained Release/Tissue-Specific Injectable Hydrogel

In order to achieve the tissue-specific and sustained delivery of siMMP-9 for diabetic wounds, Lan et al. developed a thermosensitive hybrid hydrogel biomatrix with the combination of GT/siMMP-910. In this biomatrix, they utilise the combination of PF and MC as a thermosensitive hydrogel dressing. The PF and MC percentage were adjusted to match the different desired longevities of the therapeutic substance to be released. Consistent with diabetic wound dressing interval, 7 days of sustained release were achieved without reapplication. This will assist in accelerating the wound healing process, hence elevating the patient’s quality of life [65].

High blood glucose levels in chronic or diabetic wounds are known to cause microvascular endothelial injury and diastolic vascular dysfunction. Therefore, glucose-responsive injectable hydrogels are recompensed to combat this type of wound. A novel study by Zhao et al. utilises a glucose-responsive biomatrix as an injectable hydrogel. They incorporated two main active components namely insulin and fibroblast (L929) and the hydrogels are engineered to possess a fast release at higher glucose concentration hence sanction cell proliferation in the biomatrix. An in-vivo study on diabetes-induced rats revealed that the injectable biomatrix stimulates neovascularisation and collagen deposition in the wound [66].

#### 7.2.4. Development of Multifunctional Injectable Hydrogel

Novel biomaterials are anticipated to possess a hybrid biofunction to accelerate wound healing. An in vivo study by Qu et al. sheds light on the multifunctional injectable hydrogel that exhibits the properties of antibacterial, antioxidant, and electro responsive. The hydrogel encapsulates amoxicillin as the main composition, which assists the wound healing process by preventing wound infection (inhibition of ROS in the wound area). This significantly stimulates the wound healing process with elevated tissue granulation, collagen configuration, neovascularisation, and angiogenesis in the full-thickness diabetic wound rats. Also, 2 mg amoxicillin/mL of hydrogel is able to cause wound contraction, which surpasses 18% of the commercialised film (*p* < 0.05) while the highest cumulative zone of inhibition was observed on day 3 (*p* < 0.05) against both negative and gram-positive bacteria [67].

#### 7.2.5. Development of Hypoxic Injectable Hydrogel

Injectable hydrogels with the capability to cast a hypoxic microenvironment possess a great potential to develop novel therapies for tissue regeneration. However, the relative research remains at the conceptual phase. Jin et al. chose diabetic wound as a representative injury model to explore the actual therapeutic results of tissue injury by injectable hypoxia-induced hydrogels. Briefly, adipose-derived stem cells were encapsulated in the biomatrix, which later showed an acceleration of neovascularisation and immunoregulation. It also stimulates the reconstruction of blood vessels, hair follicles, and dermal collagen matrix ushering to the recovery of the diabetes-induced wound and reconstruction of skin functions. The wound was assessed for 21 days and the study demonstrated an 80% ratio of wound closure in 14 days and 95% in 21 days [68].

#### 7.2.6. Development of Self-Healing Injectable Hydrogel

Li et al. reported a bioactive self-healing antibacterial injectable dual-network silica-based nanocomposite hydrogel scaffolds that can significantly enhance diabetic wound healing/skin tissue formation through promoting early angiogenesis without adding any bioactive factors. The nanocomposite scaffold comprises the main network of polyethylene glycol diacrylate (PEGDA) forming scaffolds with an auxiliary dynamic network between bioactive glass nanoparticles containing copper (BGNC) and sodium alginate (ALG) (PABC scaffolds). PABC scaffolds exhibit biomimetic elastomeric mechanical properties, excellent injectability, self-healing behaviour and robust broad-spectrum antibacterial activity. Importantly, PABC hydrogel significantly promoted the viability, proliferation and angiogenic ability of endothelial progenitor cells (EPCs) via in vitro. An in vivo study showed that PABC hydrogel could efficiently restore blood vessel networks through enhancing HIF-1α/VEGF expression, collagen matrix deposition in the full-thickness diabetic wound, and significantly accelerate wound healing and skin tissue regeneration. The prominent multifunctional properties and angiogenic capacity of PABC hydrogel scaffolds enable their promising applications in angiogenesis-related regenerative medicine [69].

Furthermore, Zhu et al. highlighted the benefit of fibroblasts on skin regeneration. They provided a very facile way to make tissue adhesive injectable hydrogel by mixing silica nanoparticles in an aqueous solution glycol chitosan. Hydrogel was formed due to the interaction between the nanoparticles and the polysaccharides, which has confined the latter’s movement. The gel’s lap-shear stretching force to adhere to the mouse skin pieces is up to 90 kPa allowing it to be pasted onto the wound of mice without the use of additional bandage. Whenever fibroblasts are loaded into the injectable hydrogel, it is discovered that the fibroblasts favoured the generation of micro vessels and hair follicles in the neo-skin tissue and inhibited the formation of scar [70]. Compared to bioinert silica, bioactive glass (BG) has the function of promoting angiogenesis. Kong et al. showed that the combination of BG with desferrioxamine (DFO) in an alginate-gluconolactone injectable hydrogel benefited the expression of vascular growth factors during the treatment of excisional wound on a diabetic rat model. This led to an improved prognosis of diabetic chronic skin defects [71].

Zhao et al. utilise Schiff’s based method by allying chitosan-g-polyaniline (QCSP) and benzaldehyde with the addition of functional poly (ethylene glycol)-co-poly (glycerol sebacate) as the antibacterial agents to combat feasible wound infection. The polyaniline exerts antioxidant properties hence act as free radical scavengers, which are an advantage in accelerating chronic skin defects in the diabetes-induced mouse. The results showed the synergistic effect on the expression of vascular endothelial growth factor (VEGF) and hypoxia-inducible factor-1 (HIF-1α) to promote revascularisation [71,72]. Hence, Table 3 summarized the current studies of injectable hydrogel for the management of diabetic foot ulcer.

## 8. Data Extraction Management

A bibliographic study was carried out in which the data and information of this review were obtained from multiple databases and search engines such as Google Scholar, Wiley Online Library, PubMed, ResearchGate, Elsevier, Academia and Semantic scholar until the end of February 2021. All studies addressing the injectable hydrogel with a wound healing mechanism via in vivo, in vitro, clinical studies and review articles with the result or conclusion of wound healing properties or diabetic wound healing and changes in the growth factor released were included. The search terms “injectable hydrogel”, “skin wound”, “diabetic foot ulcer”, “biomaterials”, “advanced dressings,” and “diabetic wound healing” were used in the bibliographic investigation.

## 9. Conclusions

The 3D-biomatrix system is the most preferred method as it can be utilised as a carrier for therapeutic agents, and it is easily tuneable into any desirable shape. In other words, hydrogels possess a number of advantages such as a high percentage of biocompatibility compared to conventional dressings, are able to mimic the characteristics of ECM, and are also proven to be permeable to oxygen and nutrient. The most vigorously studied are the physicochemical properties of the hydrogel for future use in the biomedical field. Due to the pain and discomfort of invasive surgery upon the implantation of pre-form hydrogel, this scientific review sheds light on injectable hydrogel to overcome the drawbacks. Injectable hydrogels can help avoid such invasive surgery and can be injected onto the defect area regardless of the shape of the targeted site and are able to be laden along with cells or pharmaceutical medicine to accelerate the proliferation of cells or to assess the delivery of growth factors.

## 10. Future Perspectives

Nowadays, 463 million adults are living with diabetes worldwide with a 51% increase in its prevalence from 2019 to 2045 to 700 million in total. Hence, the development of injectable hydrogel is being explored to combat the complication brought by the DFU using the multifunction 3D biomatrix as it is proven to assist in accelerating wound healing progress. The transition towards 3D technologies is inclined to ameliorate the current therapeutic strategies in DFU management. As discussed, it is evident that most of the biological studies on diabetes are still exploiting animal-based models and cells, which are unequivocally hard to adjust to clinical stages. Hence, it is vital to focus on human-derived biologics or biological evaluation as it is the most immediate subject to human physiology. Moreover, despite notable attempts of novel injectable hydrogel fabrication and synthesisation, several challenges remain to be addressed. The mechanical strength of hydrogels, particularly physical hydrogels require improvement, and the cytotoxicity and biocompatibility of these gels must be assessed thoroughly following recognised standards. When used as bioscaffolds or dressings, the degradation rate of the hydrogels should be adjusted in correlation with the tissue regeneration rate. Moreover, the integration of the scaffolds with host tissue and the stability of the attachment within the interface of hydrogel and native tissue should also be addressed in further studies. In delivery applications, the sustained and acceptable release profile is another factor that needs further consideration. Overall, this field needs novel hydrogel designs with tuneable properties. Future studies should focus on the potential applications of composites and hybrids to combat the burden of a chronic wound.

## Figures and Tables

**Table 1:** Advantages and complications of current wound dressings.

Advanced Dressings	Advantages	Complications
Alginate [25]	Promote homeostasis Wound fillers Low allergenic	Require secondary dressing Mistaken with pus and slough in the wound
Acrylics [26,27]	Good permeability	Low absorption Difficult to be removed
Hydrocolloids [25,27]	Aid autolytic debridement Waterproof	Prone to infection Allergy reaction Highly exudative
Foam [25,26,27]	Serve as vehicle other medication Cushioning properties	Induce maceration Frequent change
Hydrofibre [25,26,27]	High absorption Ease upon removal	Require secondary dressing Allergy reaction
Silver [25]	Antibacterial	Wound discoloration Allergy reaction

**Table 2:** Types of crosslinking method for injectable hydrogel utilization.

Crosslinking Method	Mechanism	External Stimuli
Physical	Electrostatic interactions	pH Ultrasound Electric Field Magnetic Field Temperature Light sensitivity Biomolecular species
	Hydrophobic interactions	
	Host-gest interactions	
	Van Der Waals forces	
Chemical	Diel-Alder	
	Michael addition	
	Schiff base reaction	
	Enzyme-mediation	
	Photopolymerization	

**Table 3:** Injectable hydrogels for future diabetic foot ulcer (DFU) management.

Reference	Composition	Main	Aim	Study Design	Result	Conclusion
Qu et al. 2019	N-carboxyethyl chitosan (CEC) Oxidized hyaluronic acid-graft-aniline tetramer (OHA-AT)	Amoxicillin	To develop multifunctional injectable hydrogel -anti-oxidant -antibacterial -electroactive	In vivo In vitro	Wound: (Day 15) amoxicillin loaded hydrogel (*p* < 0.05) Antimicrobial: (Day 3) Highest cumulative zone of inhibition (*p* < 0.05)	In vivo: accelerate wound healing rate than commercialized product In vitro: Effective antibacterial effect
Zhao et al. 2017	pH and Glucose Dual-Responsive	Bovine insulin	To develop sustained and pH/glucose-triggered drug release	In vivo	Wound: 58 ± 2% of collagen deposition, 2.41-fold population of red CD31-positive cells compared to control	In vivo: Infiltration of inflammation, accelerate neovascularization, collagen disposition
Qian et al. 2020	Platelet-Rich Plasma Release	Platelet-rich plasma (PRP)	To develop self-healing injectable hydrogel	In vivo In vitro	Wound: (day 21) increased the nerve density (*p* > 0.05) and (day 7) higher healing rate	In vivo: Accelerate collagen deposition, wound healing, angiogenesis, neovascularization In vitro: support human dermal fibroblast (HDF), Human Umbilical Vein Endothelial cells (HUVEC), Human Umbilical Mesenchymal Stem Cells (HUMSC) proliferation.
Jin et al., 2020	Hypoxia-Induced Conductive	Vanillin-grafted gelatin Laccase (Lac)	To develop injectable hydrogel with hypoxic microenvironment ability to assist tissue regeneration.	In vivo	Wound: HIF-1α pathway activation, 95% wound closure rate (21 days) compared to control < 75% Subcutaneous study: proangiogenic factors secretion < 0.05 (day 7)	Regulate stem cell plasticity, neovascularization, collagen deposition, hair follicle reconstruction, gene expression acceleration
Wang et al., 2019	Antibacterial exosomes	Adipose mesenchymal stem cells exosomes (AMSCs-Exo)	Evaluate angiogenesis and antibacterial ability of FHE@exo hydrogel	In vitro In vivo	HUVEC: formation of 45 vessels compared to controlled group (20 vessels), elevated alpha-smooth muscle actin (α-SMA) expression Wound: smaller wound closure, thickest granulation tissue (day 14) in the treatment group Antibacterial study: no bacterial infection during the experimental period compared to control	In vitro: accelerate proliferation, migration, angiogenesis In vivo: less scar formation, wound healing acceleration.
Chen et al. 2019	Thiolated polyethylene glycol (SH-PEG) Silver nitrate (AgNO3)	Desferrioxamine (DFO)	Evaluate angiogenesis and antibacterial abilities of DFO on HUVEC and diabetic-induced rats.	In vitro In vivo	HUVEC: extensive vascular tubule formations after treatment Wound: dry and 50% reduction compared to control (day 7) Antibacterial study: minimal intensity Staphylococcus aureus compared to control	Invitro: Show antibacterial and angiogenic capability. In vivo: Proven antibacterial and enhance angiogenesis.
Bai et al. 2020	Bone marrow mesenchymal stem cells (BM-MSCs) growth factors.	Hyaluronic acid (HA) Adipic acid dihydrazide (ADH)	Evaluate inflammatory microenvironment in diabetic induce rats	In vivo	Wound: Significantly smaller (*p* < 0.05) wound, growth factors elevate (*p* < 0.01) at day 15.	In vivo: Formation of granulation tissue, collagen deposition, nucleated cell proliferation, neovascularization
Li et al. 2020	Polyethylene glycol diacrylate (PEGDA)	Nanoparticles (copper + sodium alginate)	Evaluate angiogenic properties of hydrogel on diabetic induce mice.	In vitro In vivo	Antibacterial study: inhibit * *p* < 0.05 and ** *p* < 0.01 of Staphylococcus aureus and Escherichia coli, respectively. Wound: Peaked blood flow at day 7 with the treatment group.	In vitro: Accelerate proliferation and angiogenesis property of endothelia cells (EPCs) In vivo: Promote neovascularization, collagen deposition, and wound healing acceleration
Wang et al. 2020	Nanoezyme- Reinforced	Insulin manganese dioxide (MnO2) nanosheet	To develop multifunctional injectable hydrogel	In vivo	Wound: (Day 14) No scar tissue Antibacterial: (Day 14) nearly 100% reduction of bacterial colonies	In vivo: synergistically diminished inflammatory responses, stimulated angiogenesis, accelerated cell proliferation, promoted granulation tissue formation and extracellular matrix (ECM) deposition

## Data Availability

The data presented in this study are available on request from the corresponding author.
